# Supplementary material for: ATM Inhibition Enhances Knock-in Efficiency by Suppressing AAV-Induced Activation of Apoptotic Pathways
Source: Commun Biol. 2026 Feb 6;9:177. doi: 10.1038/s42003-026-09604-z (PMC12881585; doi:10.1038/s42003-026-09604-z)
Supplement: Supplementary file 2 — Supplementary information [file 42003_2026_9604_MOESM2_ESM.pdf]

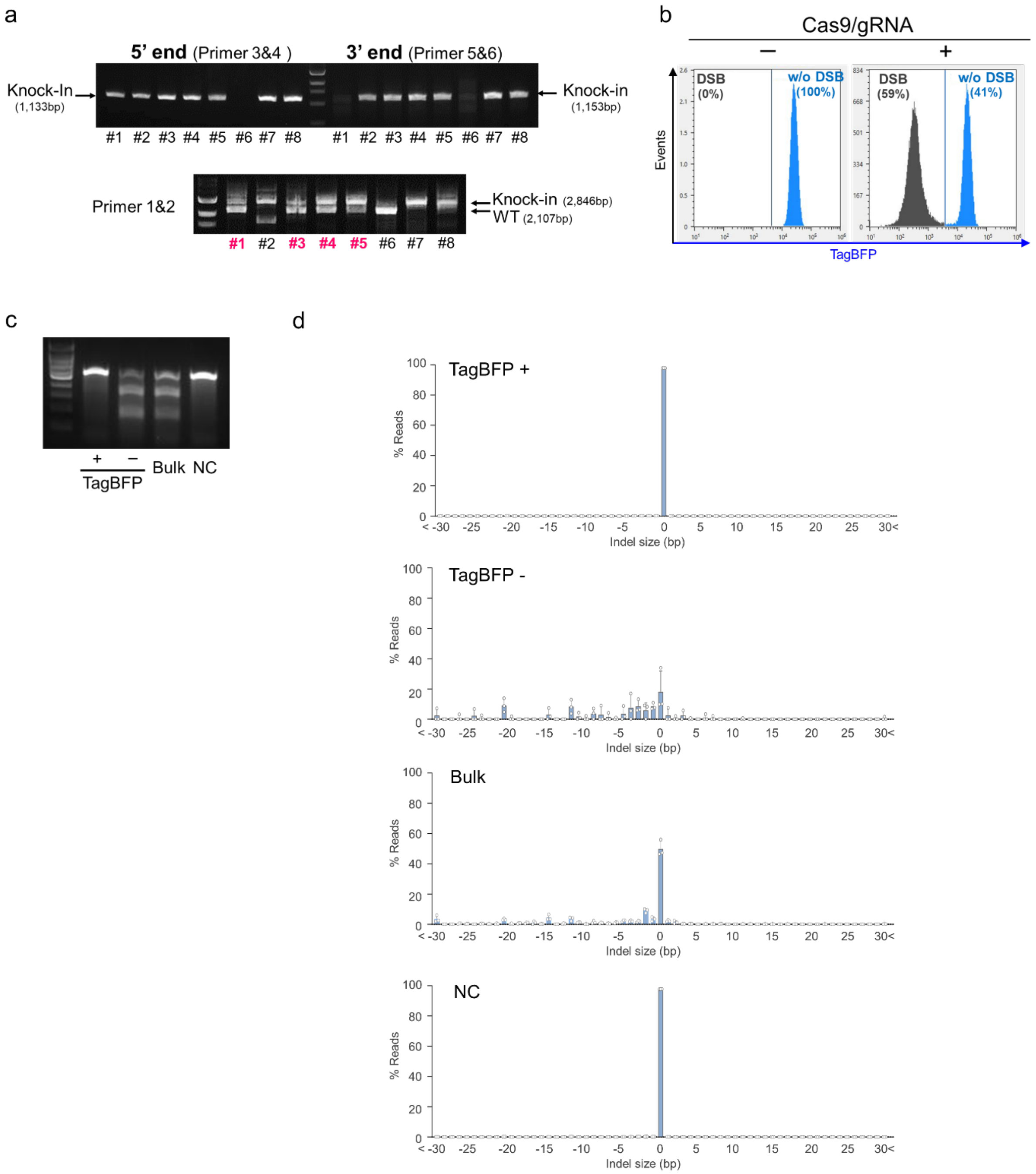

Supplementary Fig. 1| Establishment of the DSB reporter. a Agarose gel electrophoresis confirming the successful knock-in of TagBFP into the *Actb* locus. **b** Flow cytometric analysis showing a loss of TagBFP fluorescence following transfection of the plasmid vector expressing both Cas9 and gRNA. **c** Results of the Surveyor assay for cell populations sorted after genome editing. DNA fragments produced by the Surveyor nuclease were detected in the TagBFP-negative fraction, but not in the TagBFP-positive fraction, indicating that mutated cells were

present in the TagBFP-negative fraction. **d** Indel size distributions showing that the TagBFP-negative fraction contains the full spectrum of DSB-generated alleles, including in-frame mutations. “NC” indicates non-genome-edited cells, and “Bulk” indicates genome-edited but unsorted cells. Data are presented as mean ± SD. (n=3 independent experiments).

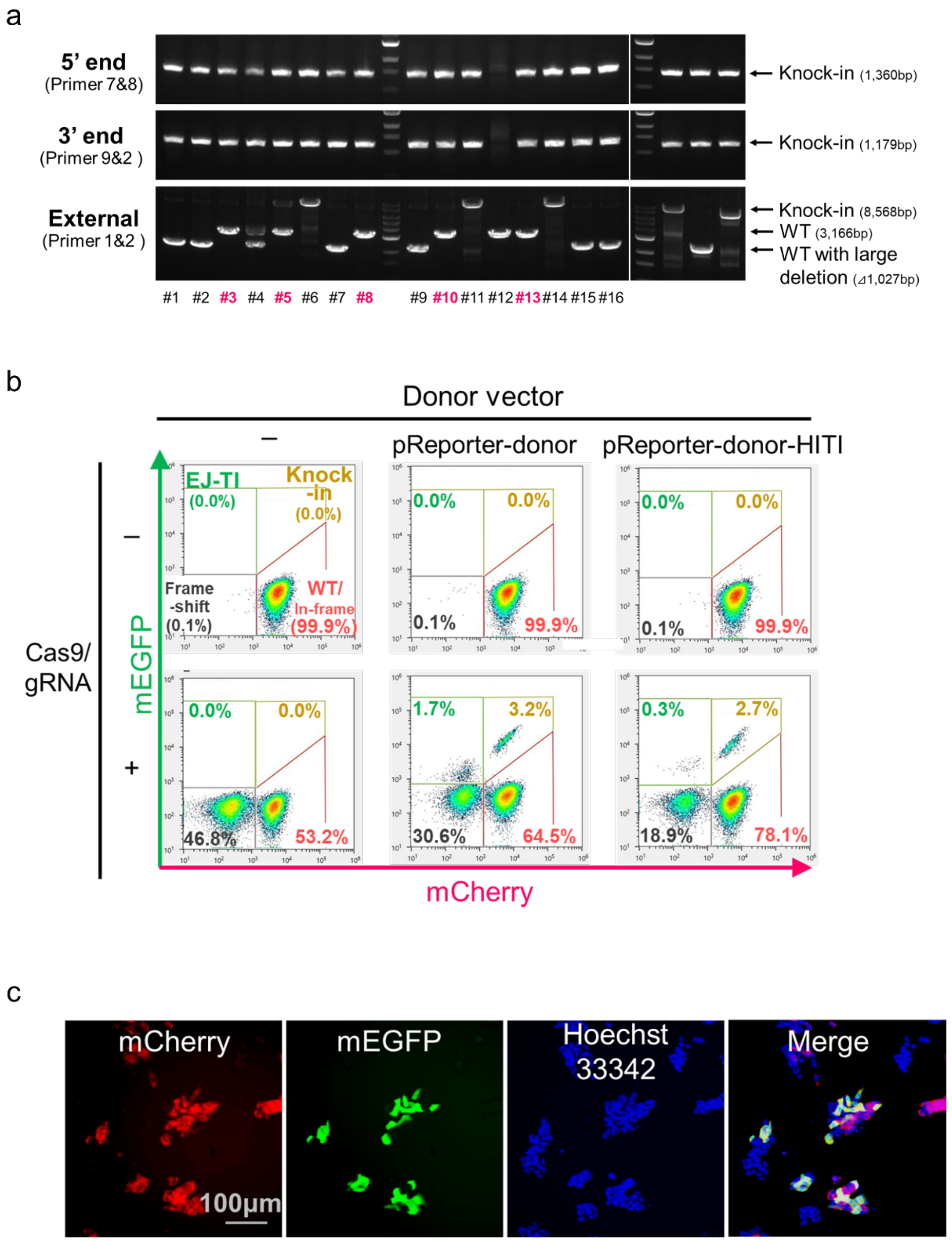

Supplementary Fig. 2| Establishment of the TI reporter. a Gel electrophoresis verifying the heterozygous integration of the targeted insertion reporter into *Actb*. **b** Flow cytometry profiling of genome-edited cells, segregating them into four distinct populations based on fluorescence: mCherry single-positive, mEGFP single-positive, double-positive, and double-negative. Genome editing was performed using a plasmid vector expressing both Cas9 and gRNA. **c** Representative

fluorescence microscopy images illustrating the expression patterns of the reporter constructs in the genome-edited cells.

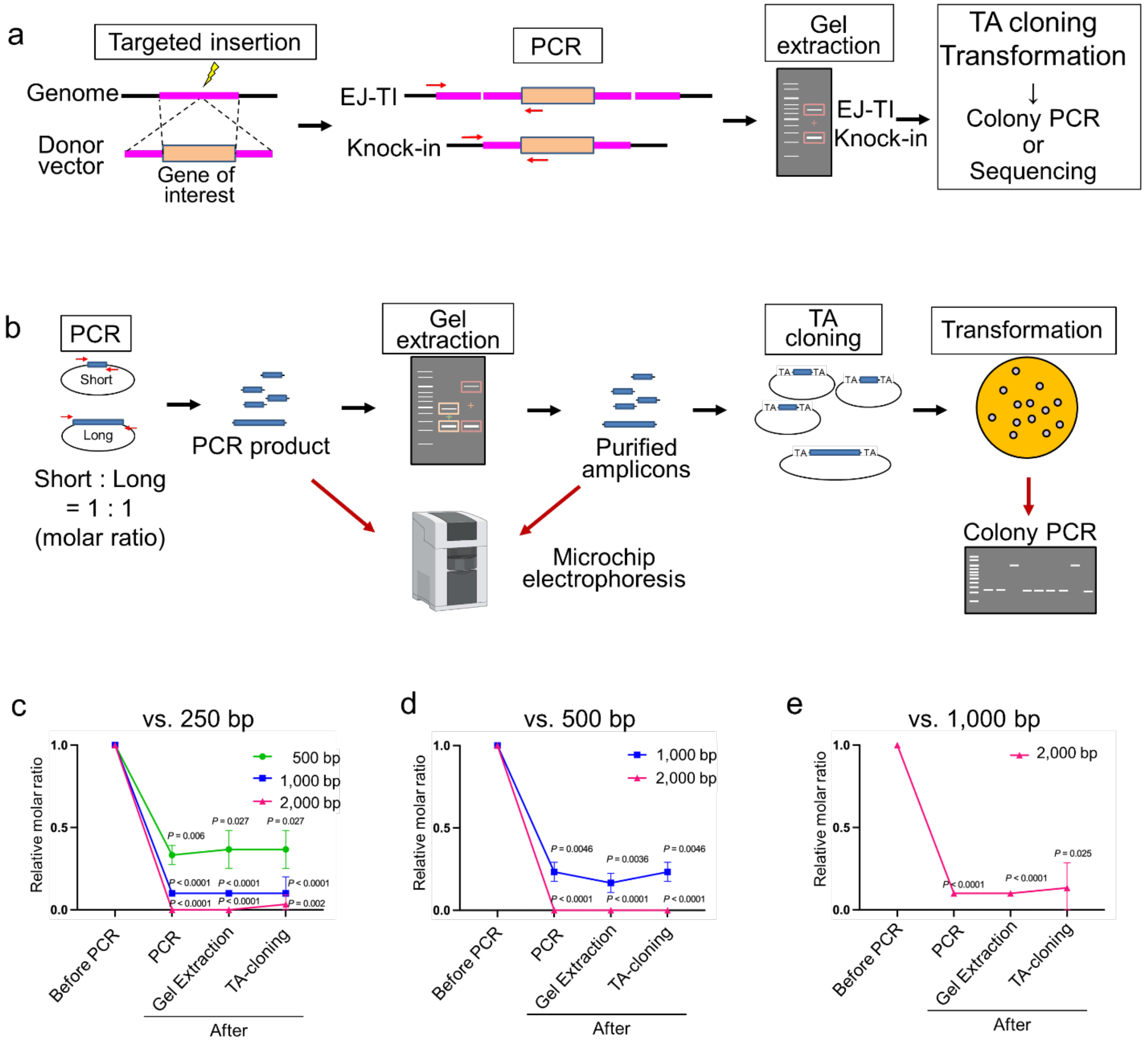

Supplementary Fig. 3| Influence of PCR product size on amplification efficiency. **a** Schematic representation of PCR- based evaluation for knock-in and EJ-TI efficiencies used in other studies. **b** Schematic representation of the method accuracy assessment for evaluating the targeted insertion efficiencies described in (a). Relative molar ratio between short and long PCR products after amplification, gel purification, and TA cloning. The microchip electrophoresis illustration was created using BioRender.com. **c** 250 bp vs. 500–2,000 bp, **d** 500 bp vs. 1,000–2,000 bp, and **e** 1,000 bp vs. 2,000 bp. Data are presented as mean ± SD. (n=3 independent experiments). Tukey's multiple comparisons tests were conducted.

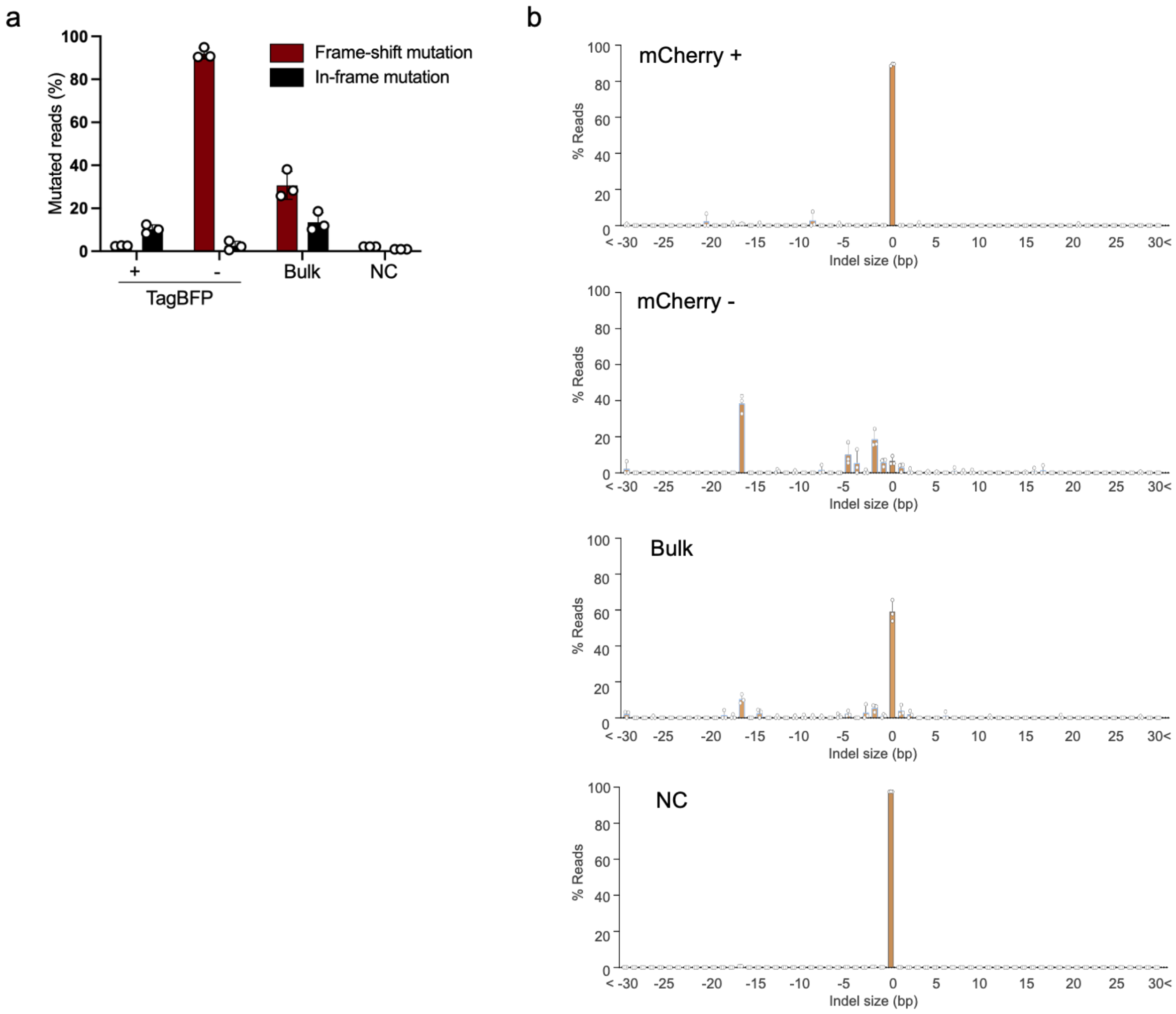

Supplementary Fig. 4| mCherry detected only frame-shift mutations. a Results of deep sequencing after generating DSB in mCherry. mCherry detected frame-shift mutations, but not in-frame mutations. “NC” indicates non-genome-edited cells, and “Bulk” indicates genome-edited but unsorted cells. **b** Indel-size distributions for the same fractions. Data are presented as mean ± SD. (n=3 independent experiments).

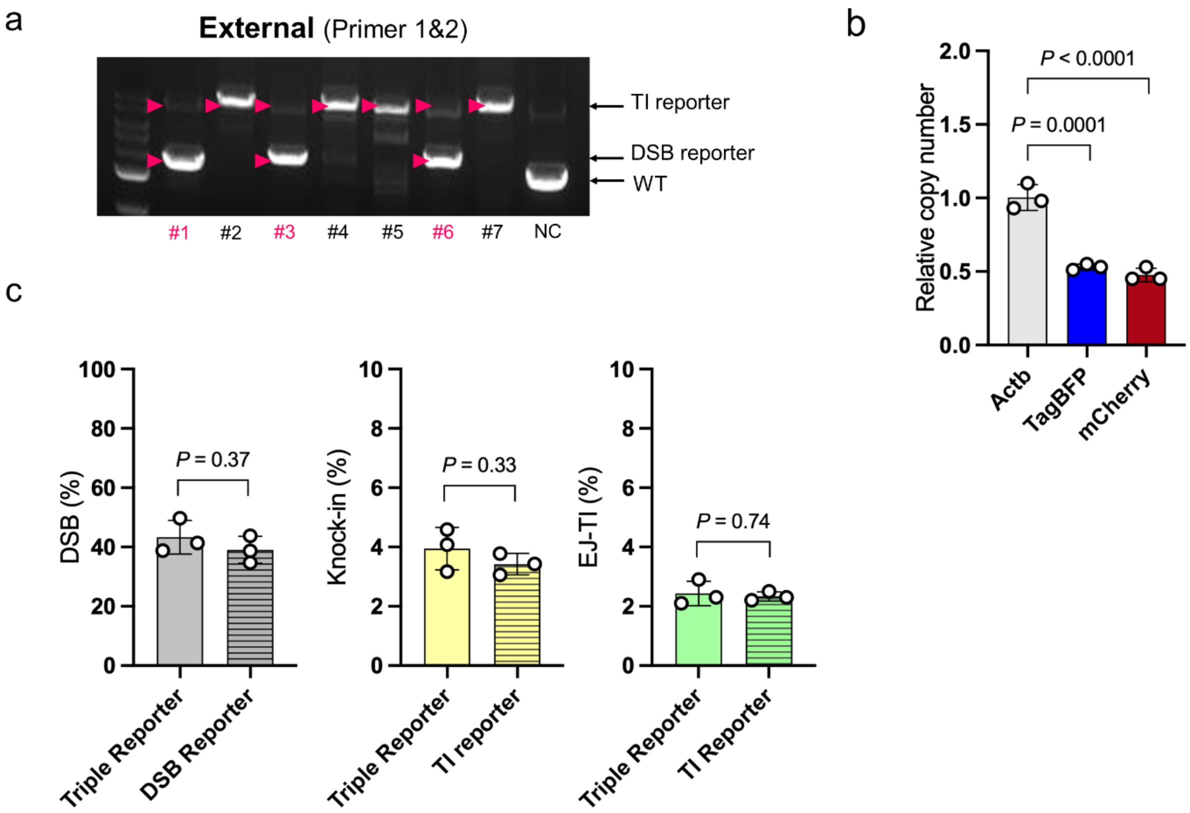

Supplementary Fig. 5| Validation of TI reporter. a PCR validation of the double-strand break (DSB) and targeted insertion (TI) reporter integration. Gel electrophoresis of PCR products amplified using external primers (Primer 1 & 2) confirmed the presence of DSB and TI reporters in multiple clones. Arrowheads denote the positive integration of reporter constructs. Notably, clones #1, #3, and #6 display integrations of both DSB and TI reporters. WT: wild-type allele, NC: negative control. **b** qPCR results showing that the copy numbers of Actb, TagBFP, and mCherry were 2:1:1, respectively, indicating that each cell harbors a single copy of the reporter cassette. Data are presented as mean ± SD. (n=3 independent experiments). Tukey's multiple comparisons tests were conducted. **c** The combination of DSB and TI reporters did not alter the detection ability of each event. Comparisons between the triple reporter and DSB or TI reporters showed no change in the respective efficiencies. Data are presented as mean ± SD. (n=3 independent experiments). Unpaired *t*-tests were conducted.

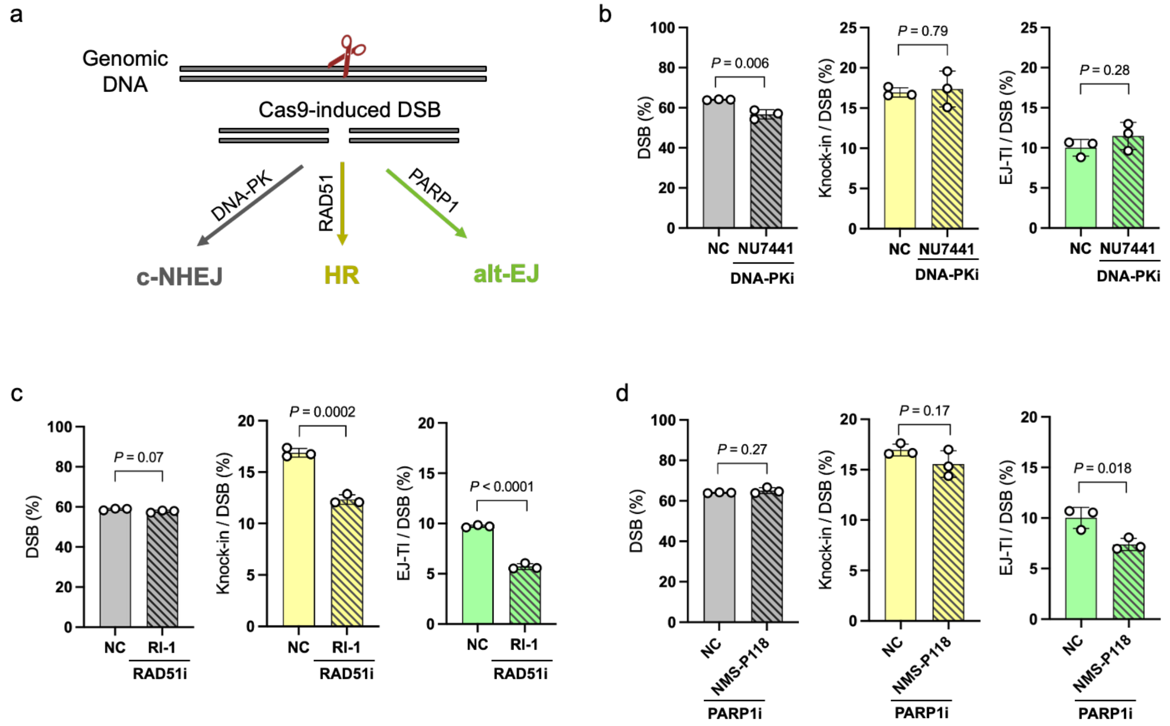

Supplementary Fig. 6| Analysis of repair pathways involved in targeted insertion using a plasmid donor vector. a Key factors involved in double-strand break (DSB) repair pathways. DNA-PK is a major component of classical non-homologous end-joining (c-NHEJ), RAD51 is essential for homologous recombination (HR), and PARP1 is involved in alternative end-joining (Alt-EJ). **b–d** Genome editing using a plasmid donor vector following treatment with inhibitors of **b** DNA-PK (10 μM NU7441), **c** RAD51 (30 μM RI-1), or **d** PARP1 (30 μM NMS-P118). Data are presented as mean ± SD. (n=3 independent experiments). Unpaired *t*-tests were conducted.

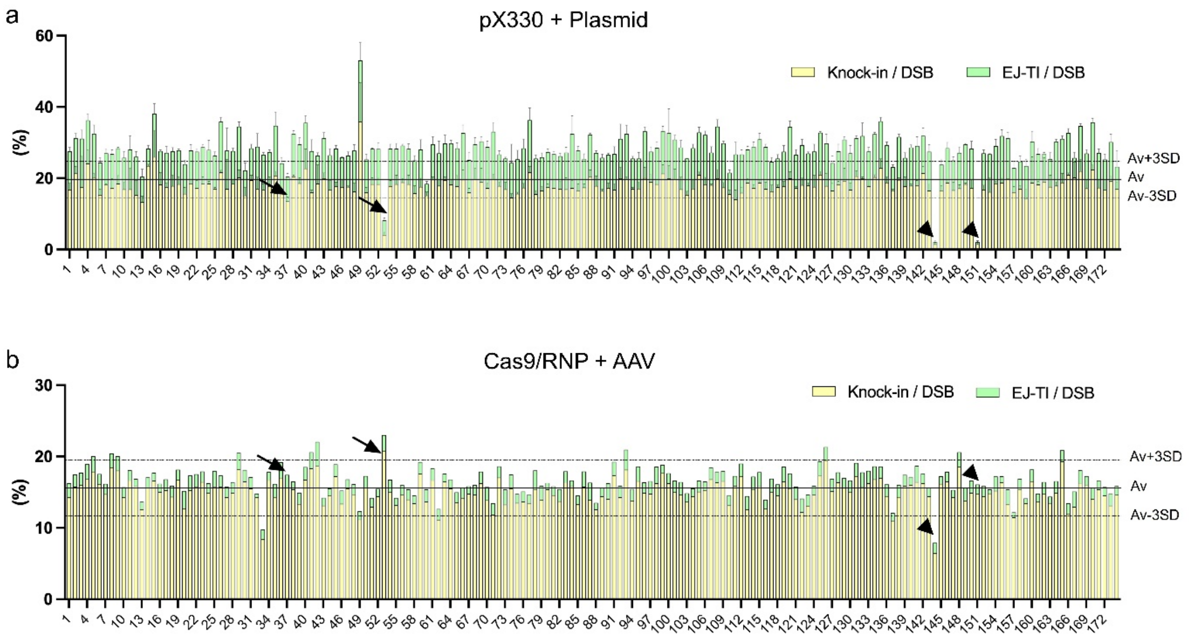

Supplementary Fig. 7| Drug screening results using plasmid or AAV donor DNA. a Screening results obtained with a plasmid donor DNA together with a plasmid expressing Cas9 and gRNA. ATM inhibitors (arrow) markedly reduced knock-in efficiency (>3 SD lower than the mean). Data are presented as mean ± SD (n = 3 independent experiments). **b** Screening results obtained with an AAV donor DNA and Cas9–RNP. ATM inhibitors (arrow) increased or did not affect knock-in efficiency, whereas ATR inhibitors (arrowhead) markedly reduced it (>3 SD lower than the mean).

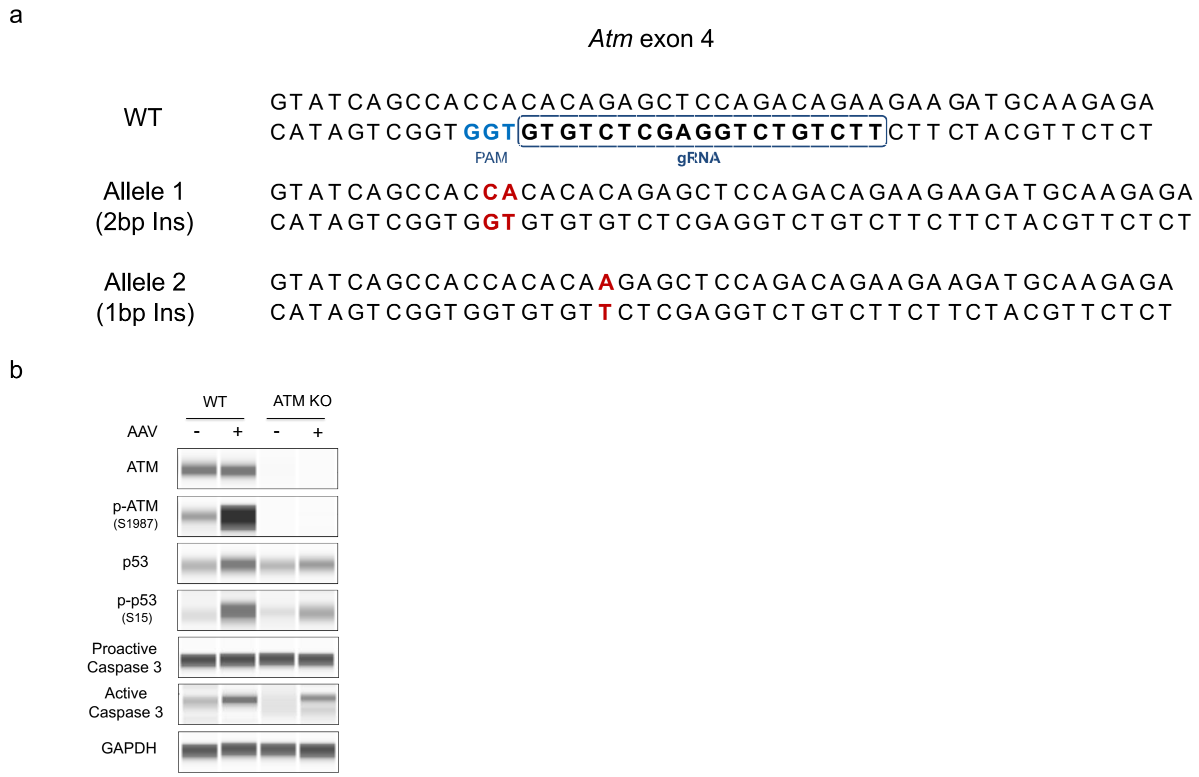

Supplementary Fig. 8| Establishment and functional validation of ATM-knockout reporter ES cells. a Target site in *Atm* exon 4 and Sanger sequencing of a single-cell clone showing biallelic frameshift insertions (+2 bp and +1 bp; inserted bases in red). **b** Western blot analysis showing that AAV-induced phosphorylation of p53 and activation of caspase 3 were attenuated in ATM-knockout cells compared with wild-type cells, showing a similar but weaker trend than pharmacologic inhibition.

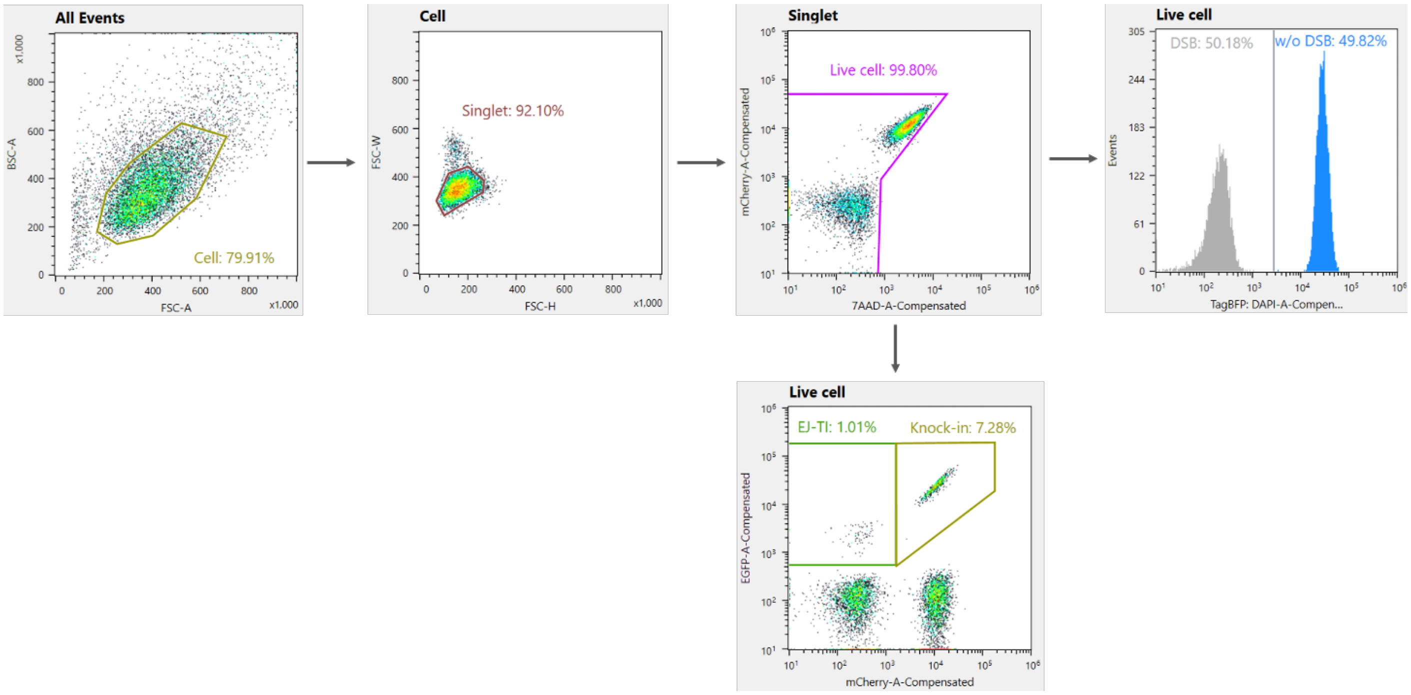

Supplementary Fig. 9| Gating strategy to detect DSB, EJ-TI, and knock-in in triple-reporter cells. Cells were first gated to define the main population, after which singlets were selected by FSC-W/FSC-H doublet exclusion. Live cells were then identified using 7-AAD. Within the live singlet population, TagBFP-negative cells were defined as cells harboring DSBs. Targeted insertion events were identified as mEGFP^{+} mCherry^{+} cells, whereas EJ-TI events were identified as mEGFP^{+} mCherry^{-} cells.

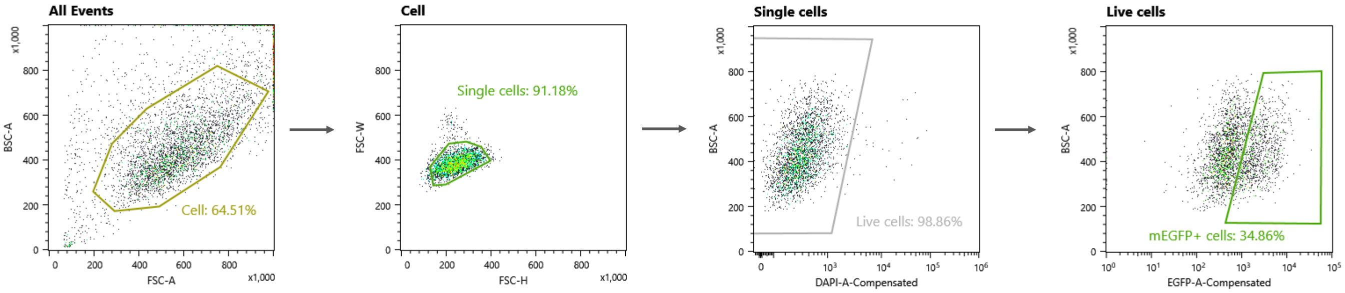

Supplementary Fig. 10| Gating strategy to detect the knock-in in HEK293T cells. Cells were first gated to define the main population, after which singlets were selected by FSC-W/FSC-H doublet exclusion. Live cells were then identified using DAPI. Within the live singlet population, mEGFP$^+$ cells were classified as knock-in events.

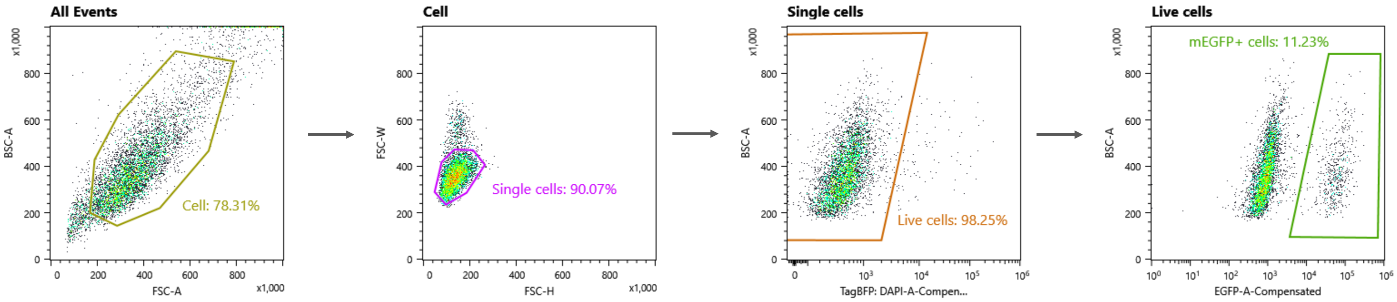

Supplementary Fig. 11| Gating strategy to detect the knock-in in mouse mesenchymal stromal cells. Cells were first gated to define the main cell population, after which singlets were selected by FSC-W/FSC-H doublet exclusion, followed by identification of live cells using DAPI. Within the live singlet population, mEGFP^{+} cells were classified as knock-in events.

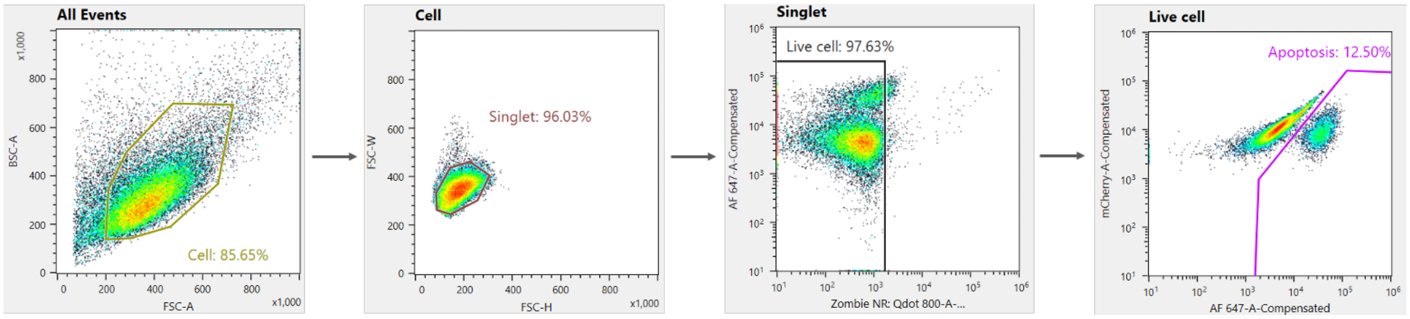

Supplementary Fig. 12| Gating strategy to detect apoptosis in triple-reporter cells. Cells were first gated to define the main cell population, after which singlets were selected by FSC-W/FSC-H doublet exclusion, followed by identification of live cells using Zombie NIR. Within the live singlet population, AF647^{+} cells were classified as apoptotic cells.

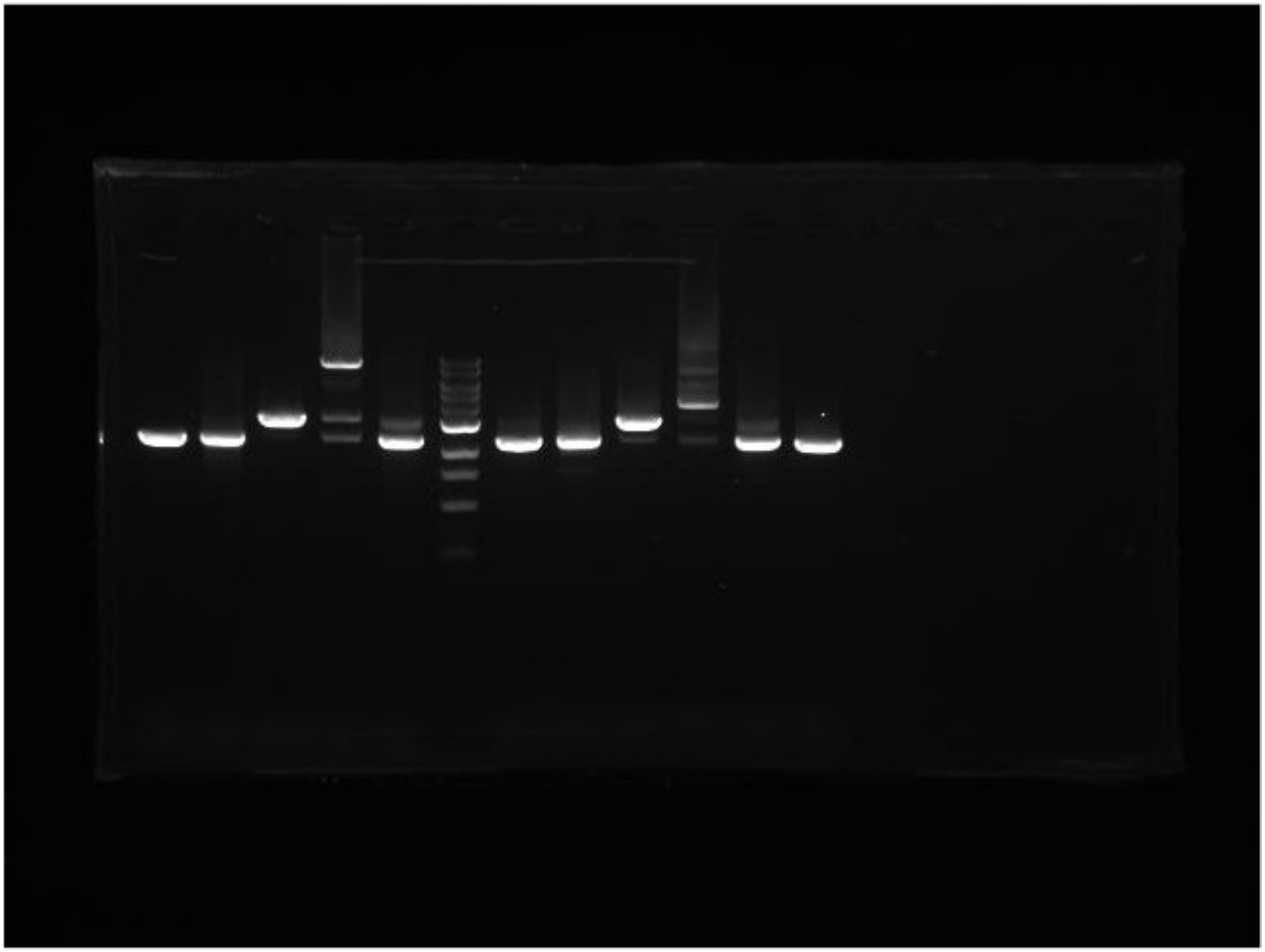

Supplementary Fig. 13| Full, uncropped PCR gel image. The original PCR gel image corresponding to the cropped panel shown in Fig. 1f is presented.

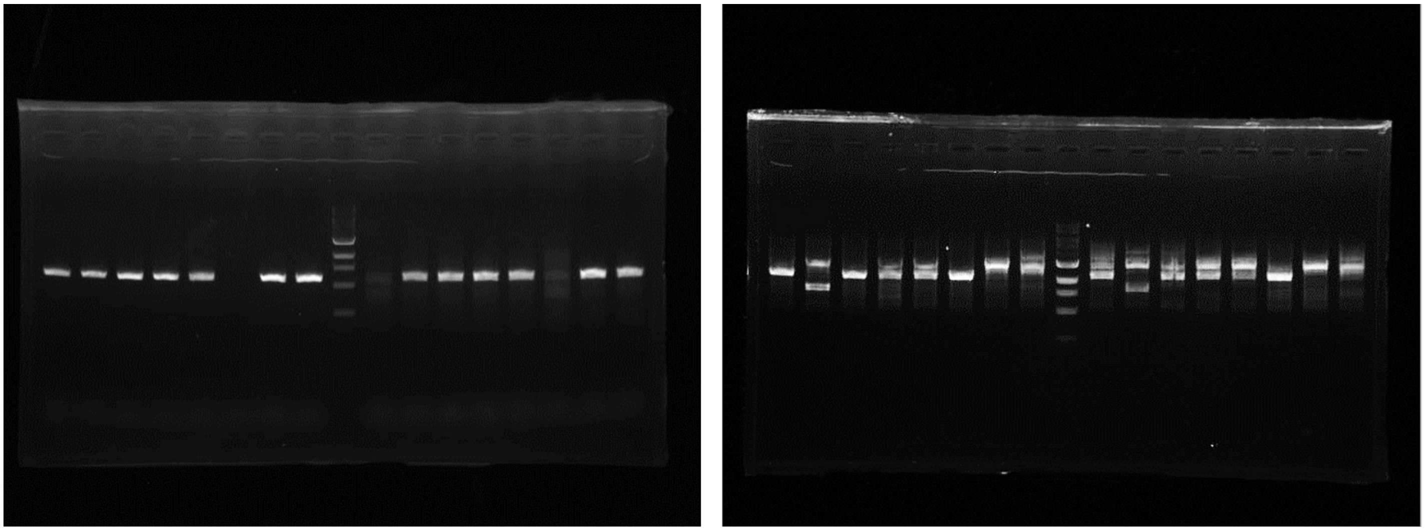

Supplementary Fig. 14| Full, uncropped PCR gel images. The original PCR gel images corresponding to the cropped panel shown in Supplementary Fig. 1a are presented.

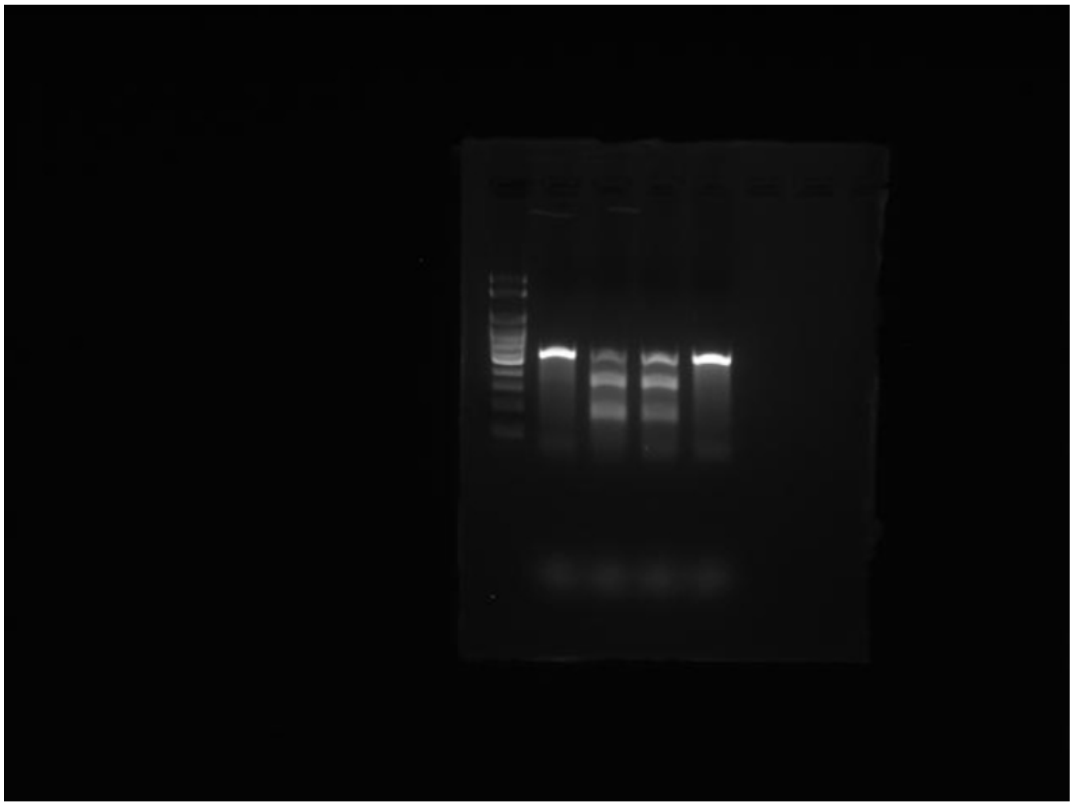

Supplementary Fig. 15| Full, uncropped PCR gel image. The original PCR gel image corresponding to the cropped panel shown in Supplementary Fig. 1c is presented.

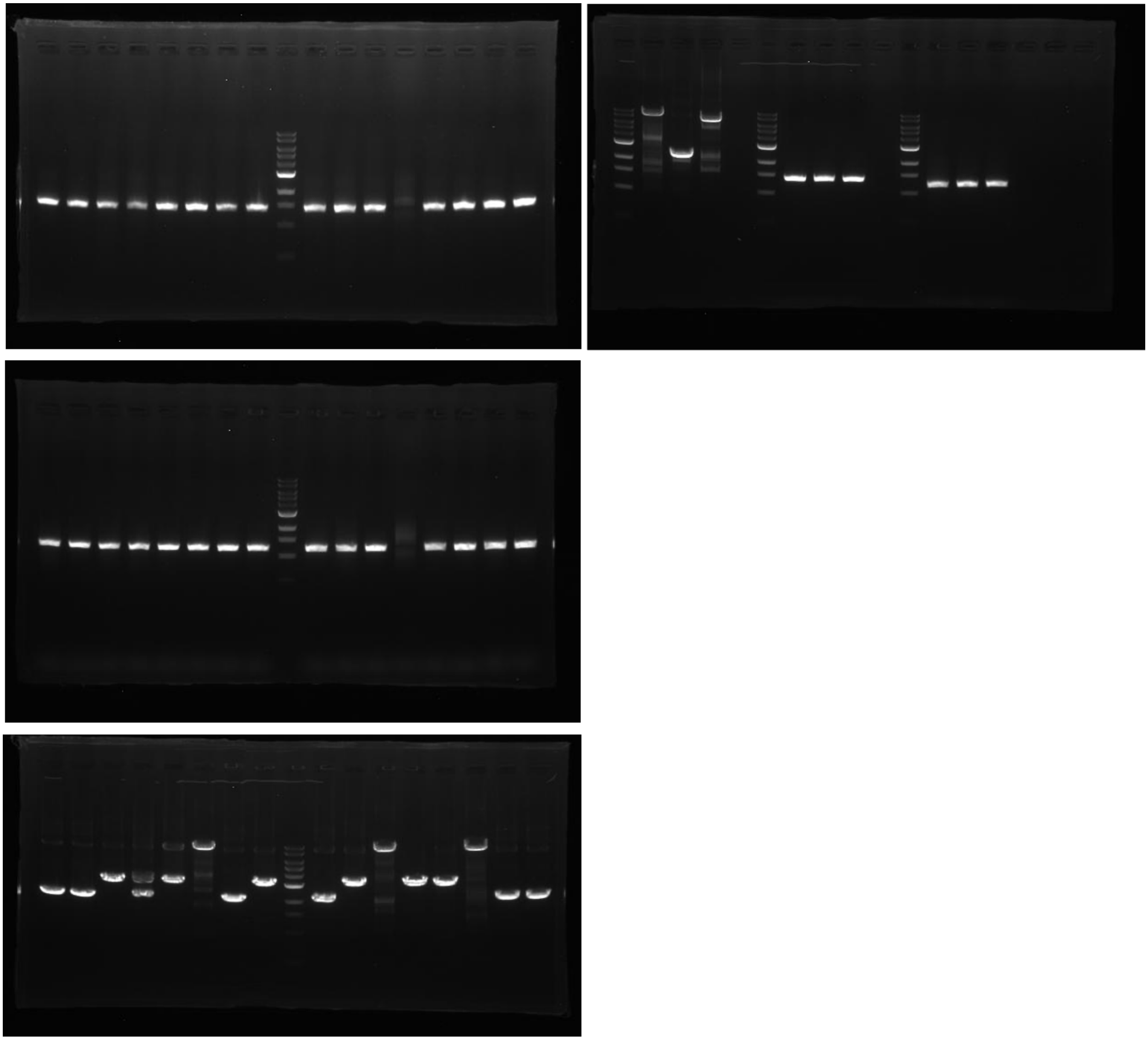

Supplementary Fig. 16| Full, uncropped PCR gel images. The original PCR gel images corresponding to the cropped panel shown in Supplementary Fig. 2a are presented.

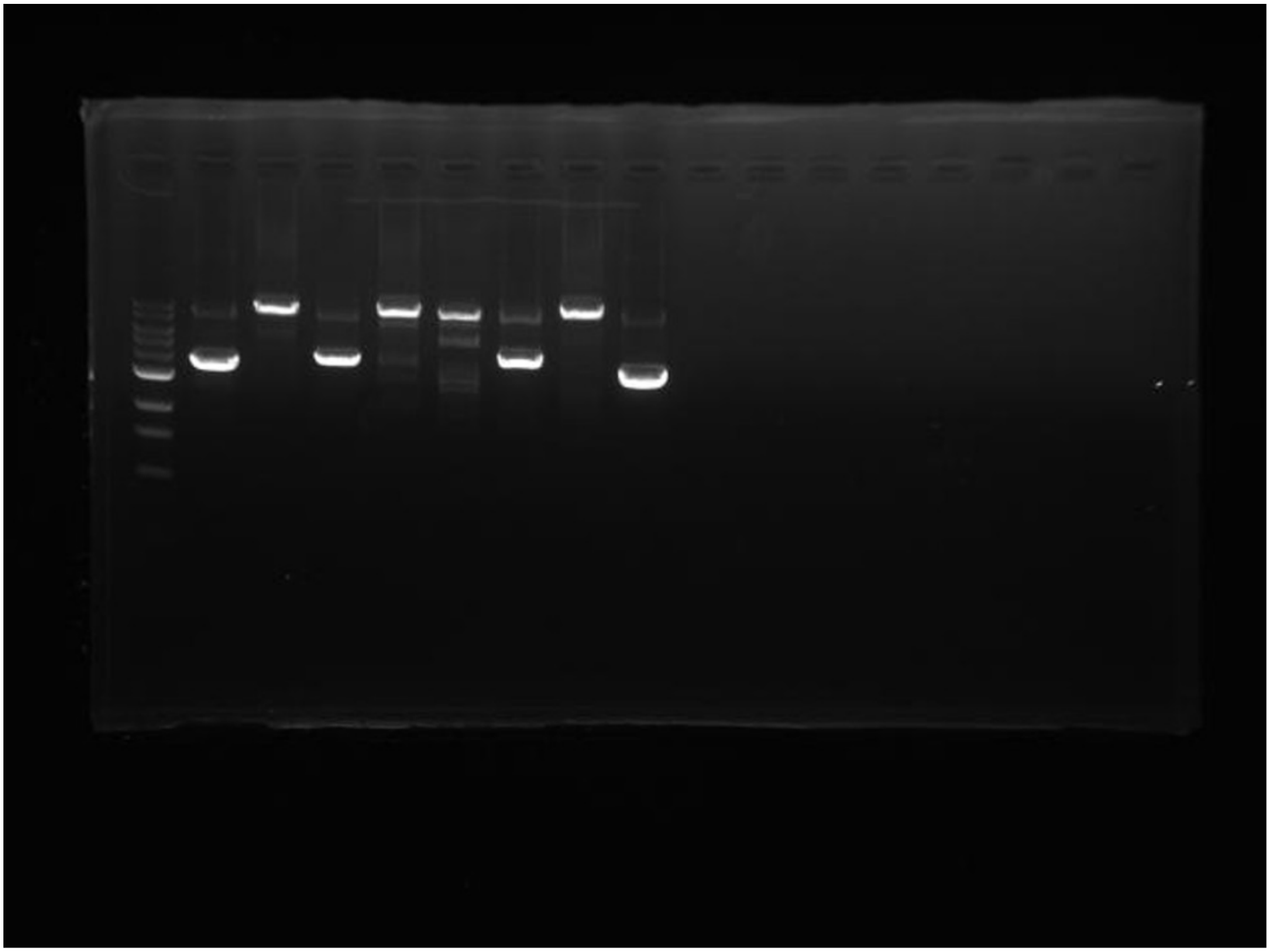

Supplementary Fig. 17| Full, uncropped PCR gel image. The original PCR gel image corresponding to the cropped panel shown in Supplementary Fig. 5a is presented.

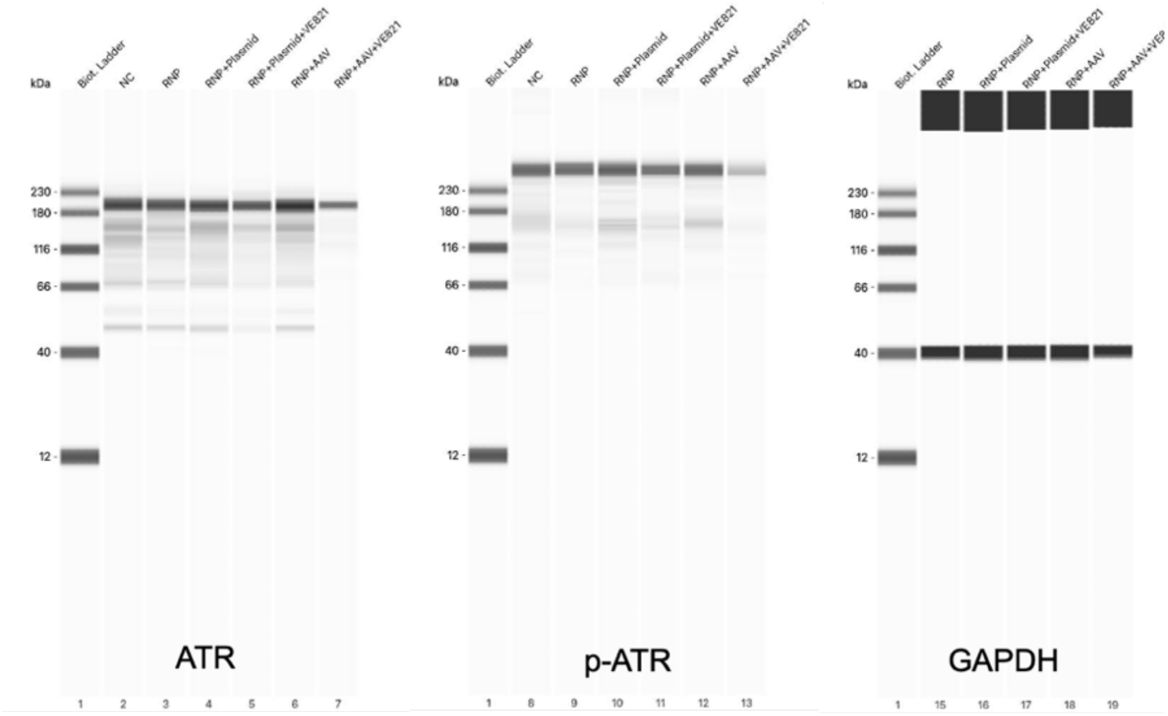

Supplementary Fig. 18| Full, uncropped Simple Western images. The original Simple Western images corresponding to the cropped panel shown in Fig. 2a are presented.

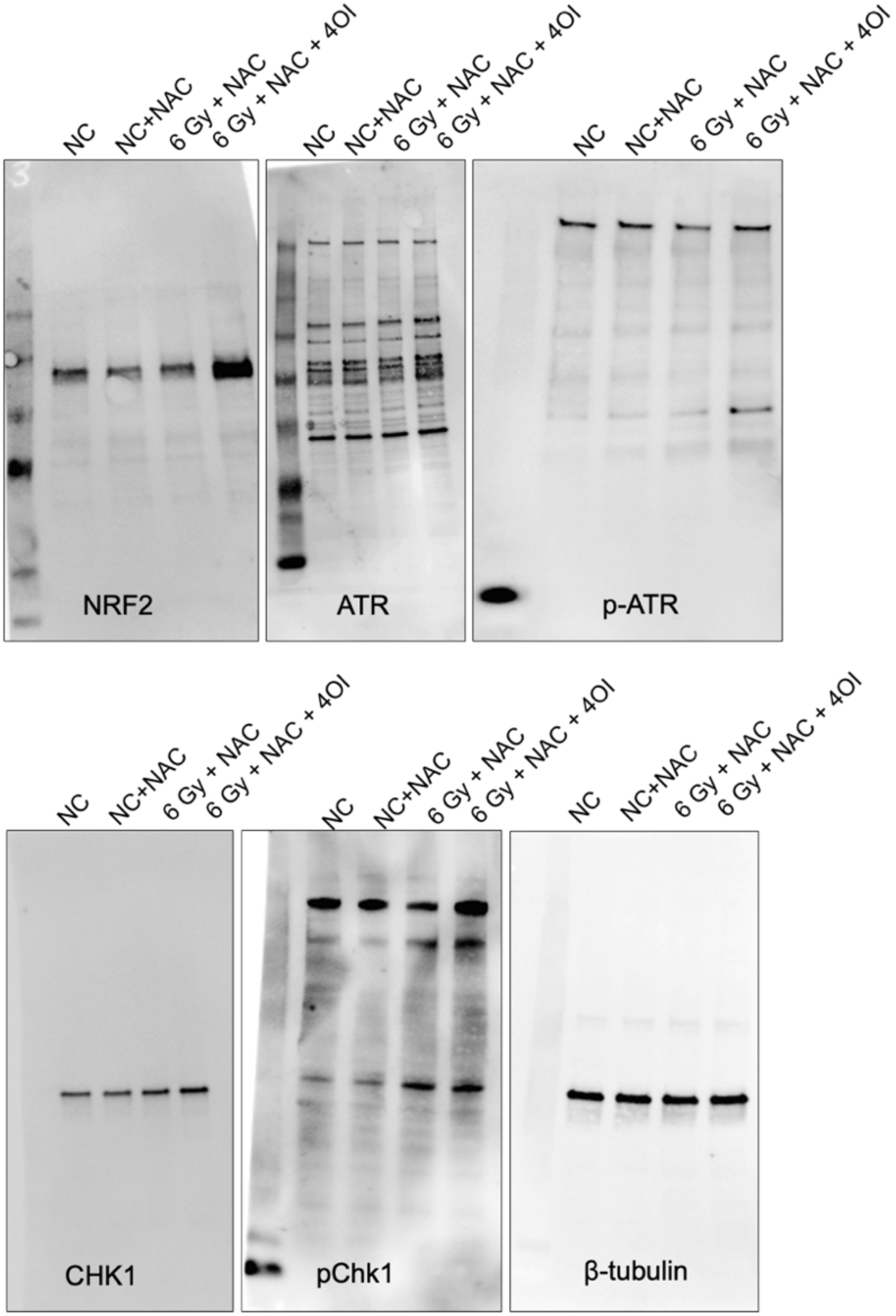

Supplementary Fig. 19| Full, uncropped western blot images. The original western blot images corresponding to the cropped panel shown in Fig. 2e are presented.

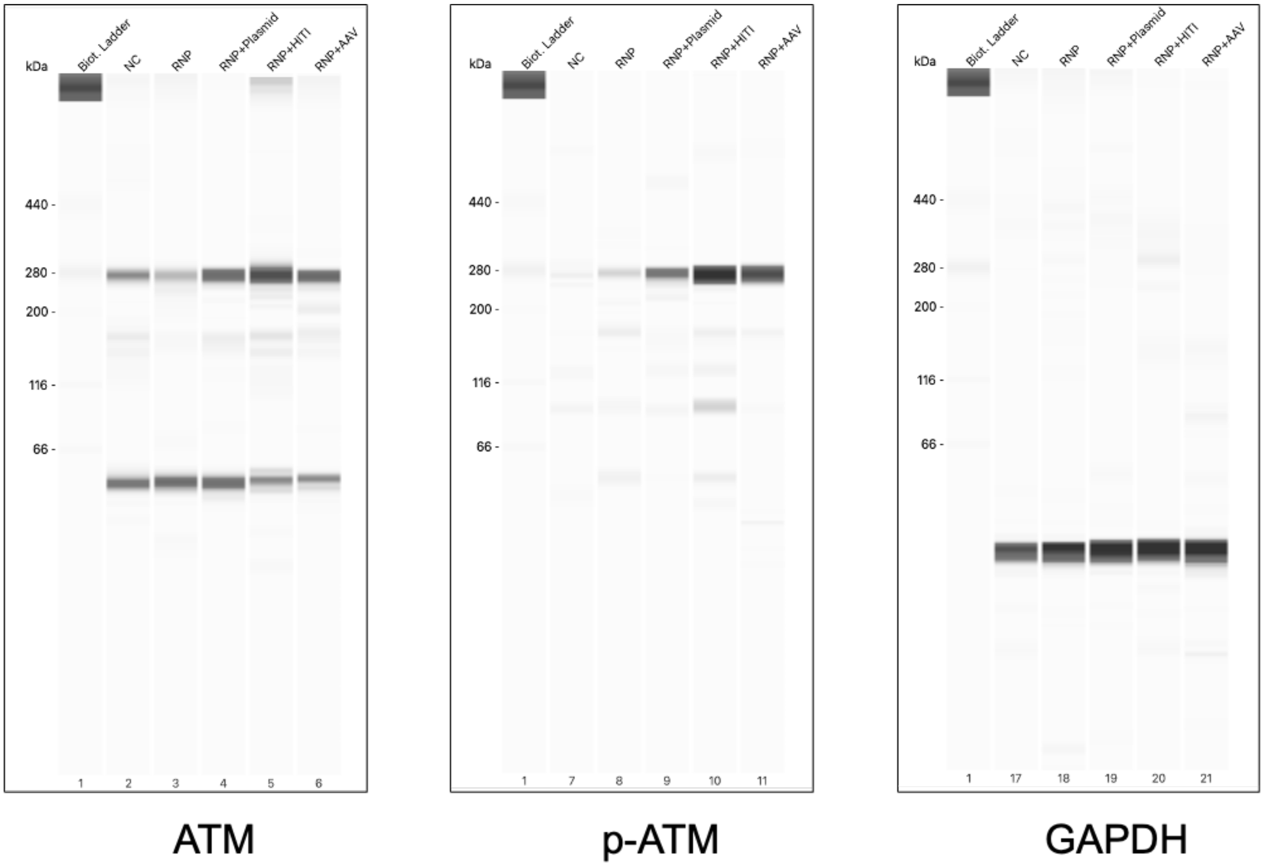

Supplementary Fig. 20| Full, uncropped Simple Western images. The original Simple Western images corresponding to the cropped panel shown in Fig. 5a are presented.

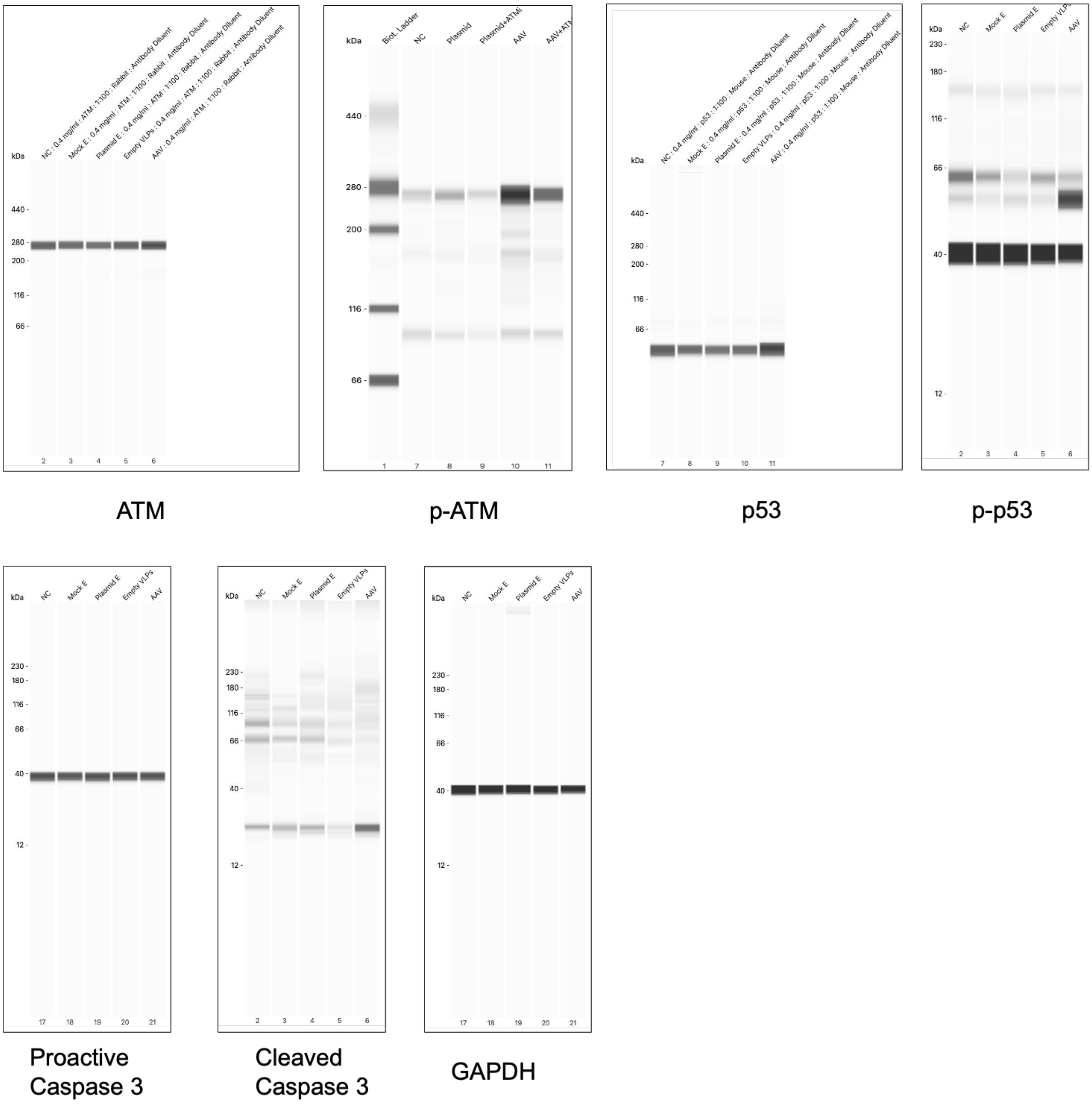

Supplementary Fig. 21| Full, uncropped Simple Western images. The original Simple Western images corresponding to the cropped panel shown in Fig. 5b are presented.

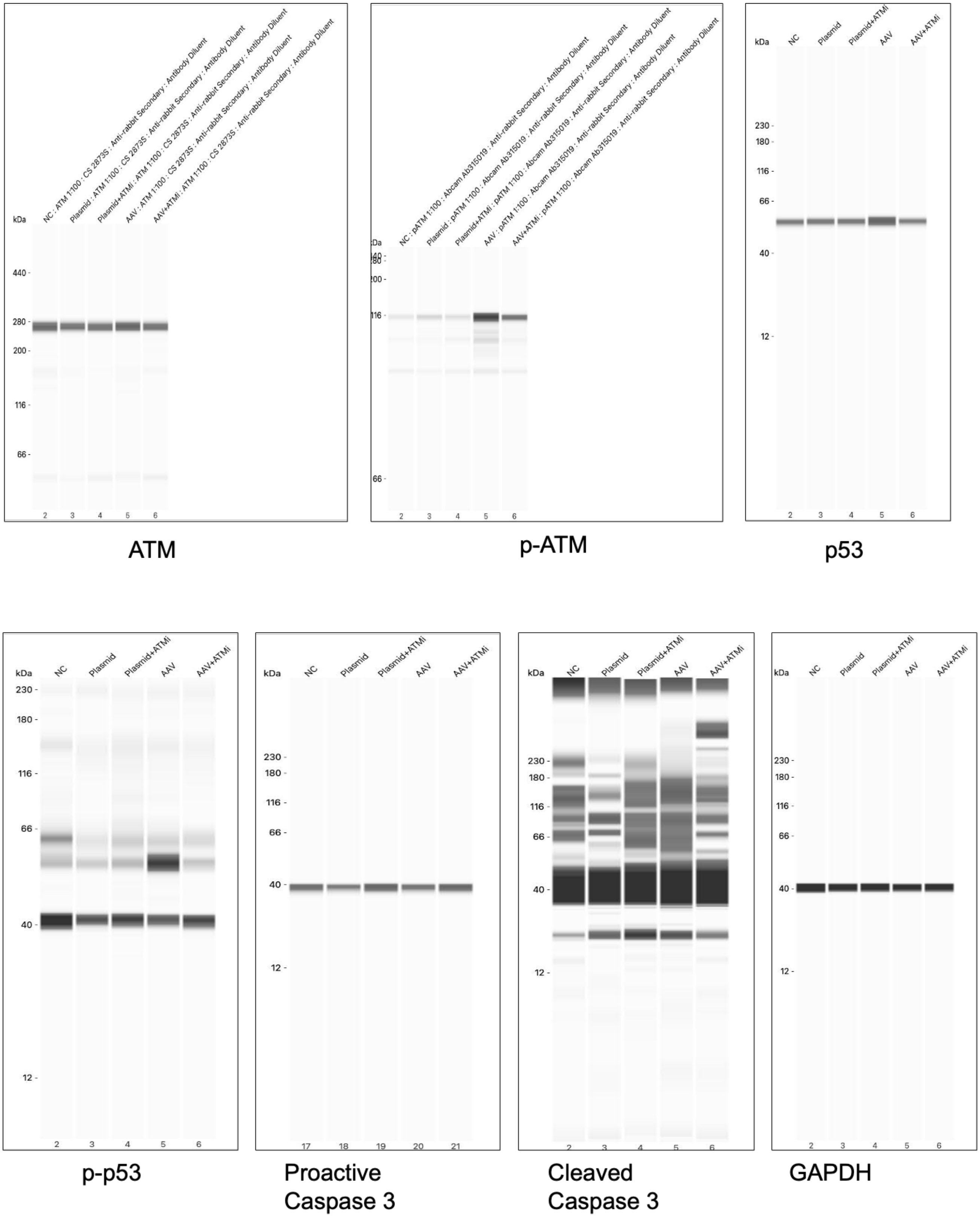

Supplementary Fig. 22| Full, uncropped Simple Western images. The original Simple Western images corresponding to the cropped panel shown in Fig. 5d are presented.

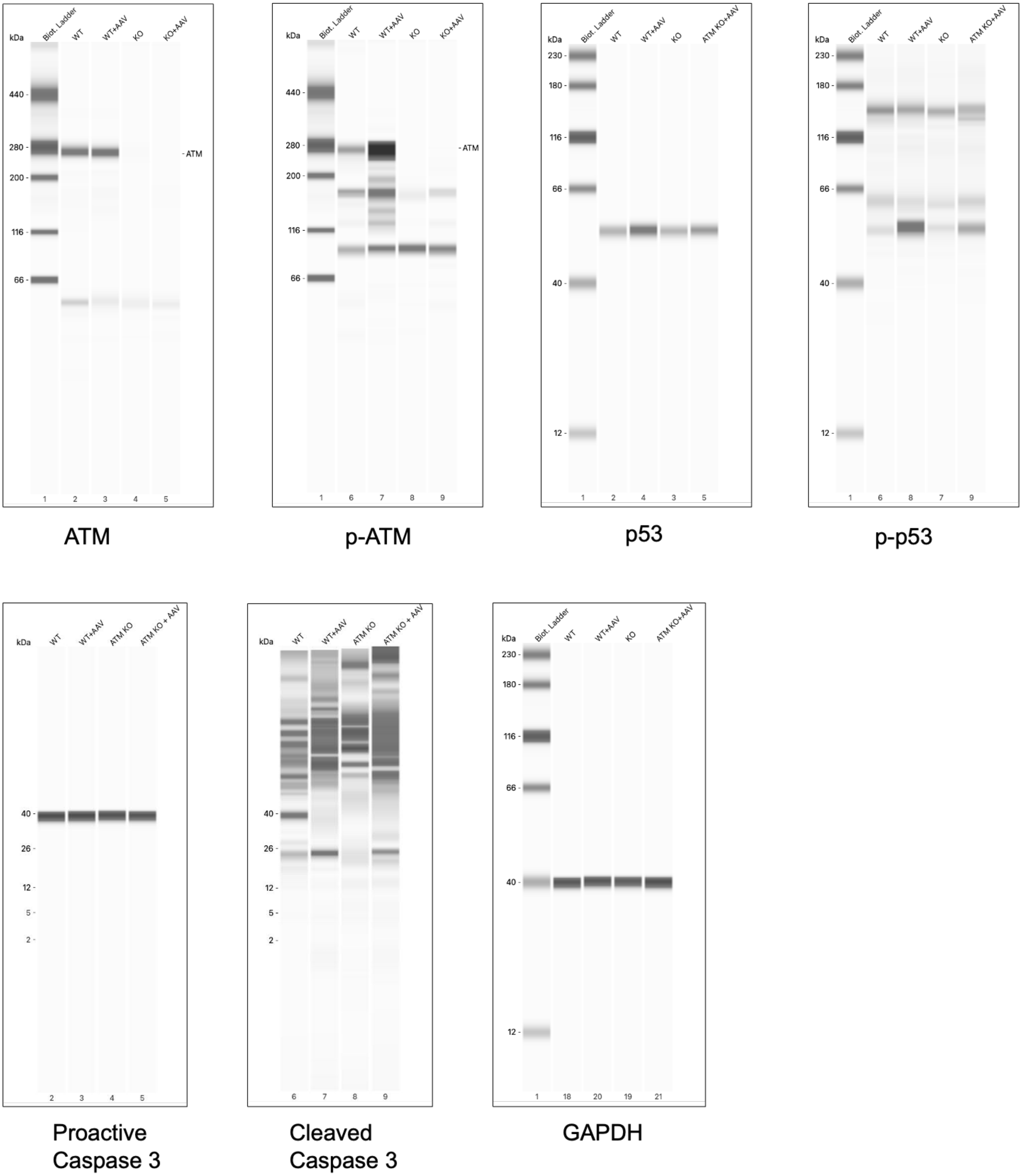

Supplementary Fig. 23| Full, uncropped Simple Western images. The original Simple Western images corresponding to the cropped panel shown in Supplementary Fig. 8b are presented.

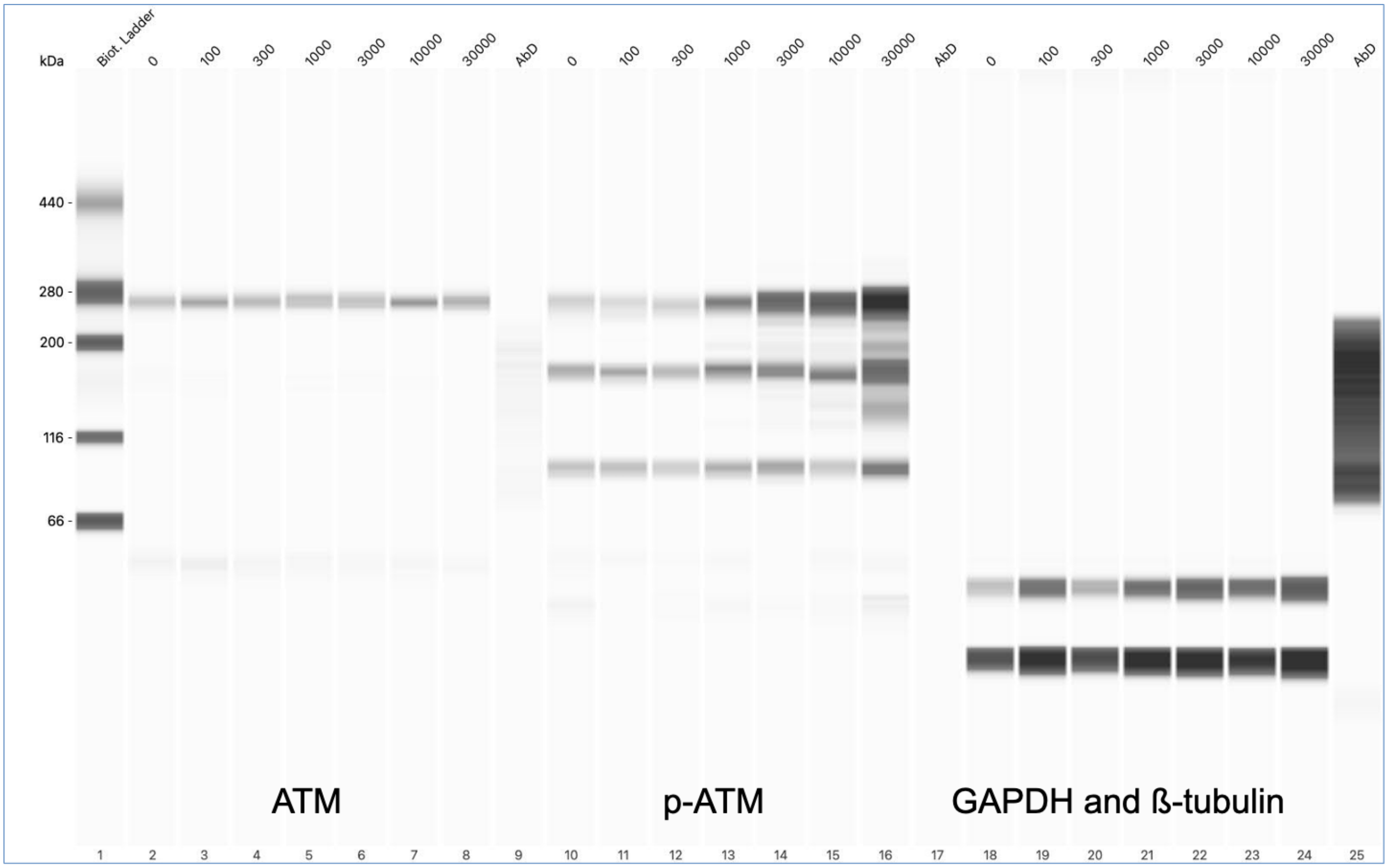

Supplementary Fig. 24| Full, uncropped Simple Western images. The original Simple Western images corresponding to the cropped panel shown in Fig. 6d are presented.

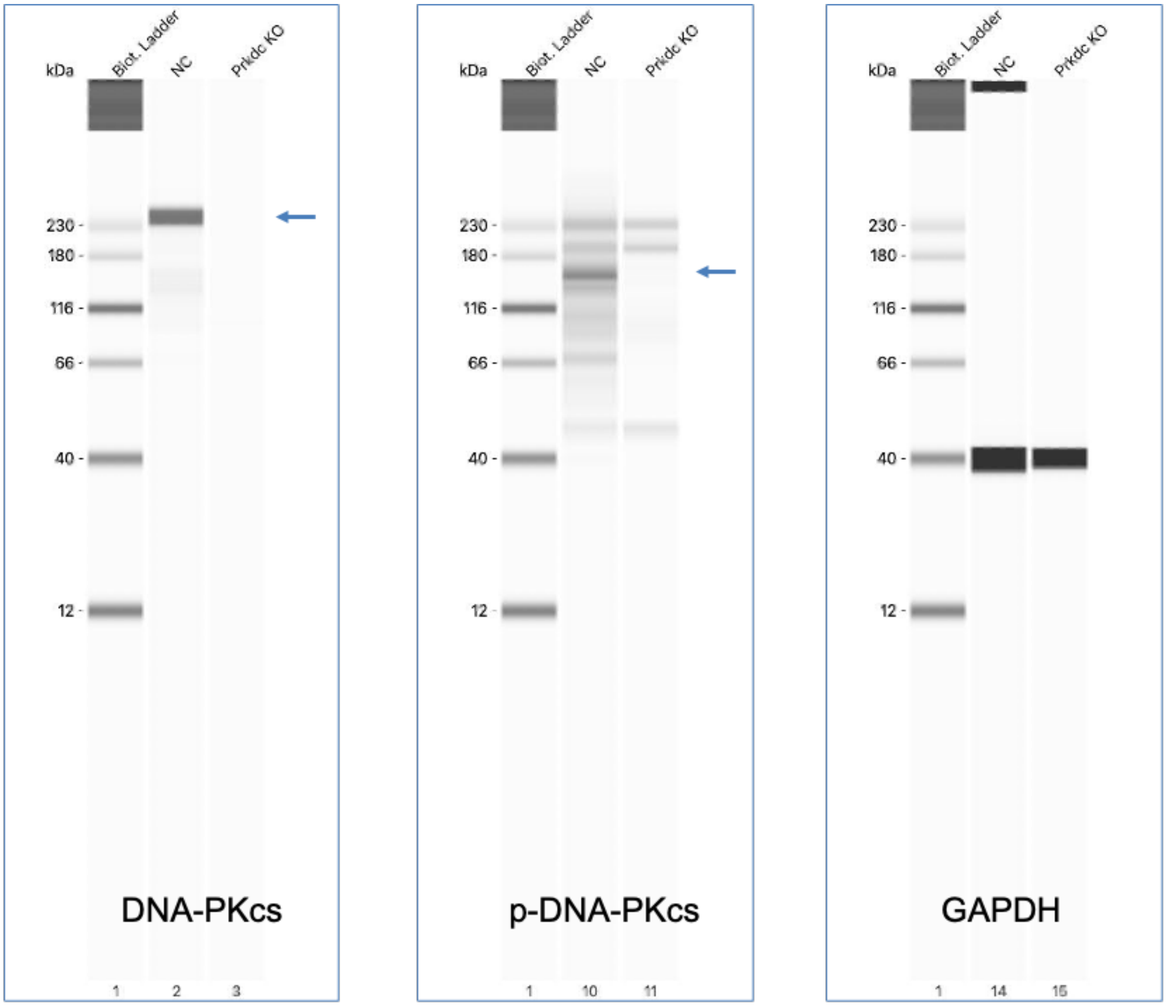

Supplementary Fig. 25| Full, uncropped Simple Western images used to determine the molecular weight of p-DNA-PKcs. Because detection of p-DNA-PKcs was unclear, protein extracts from Prkdc^{-}/$^{-}$ mouse cells were used. The arrow indicates the p-DNA-PKcs band detected in WT cells but absent in Prkdc^{-}/$^{-}$ cells.

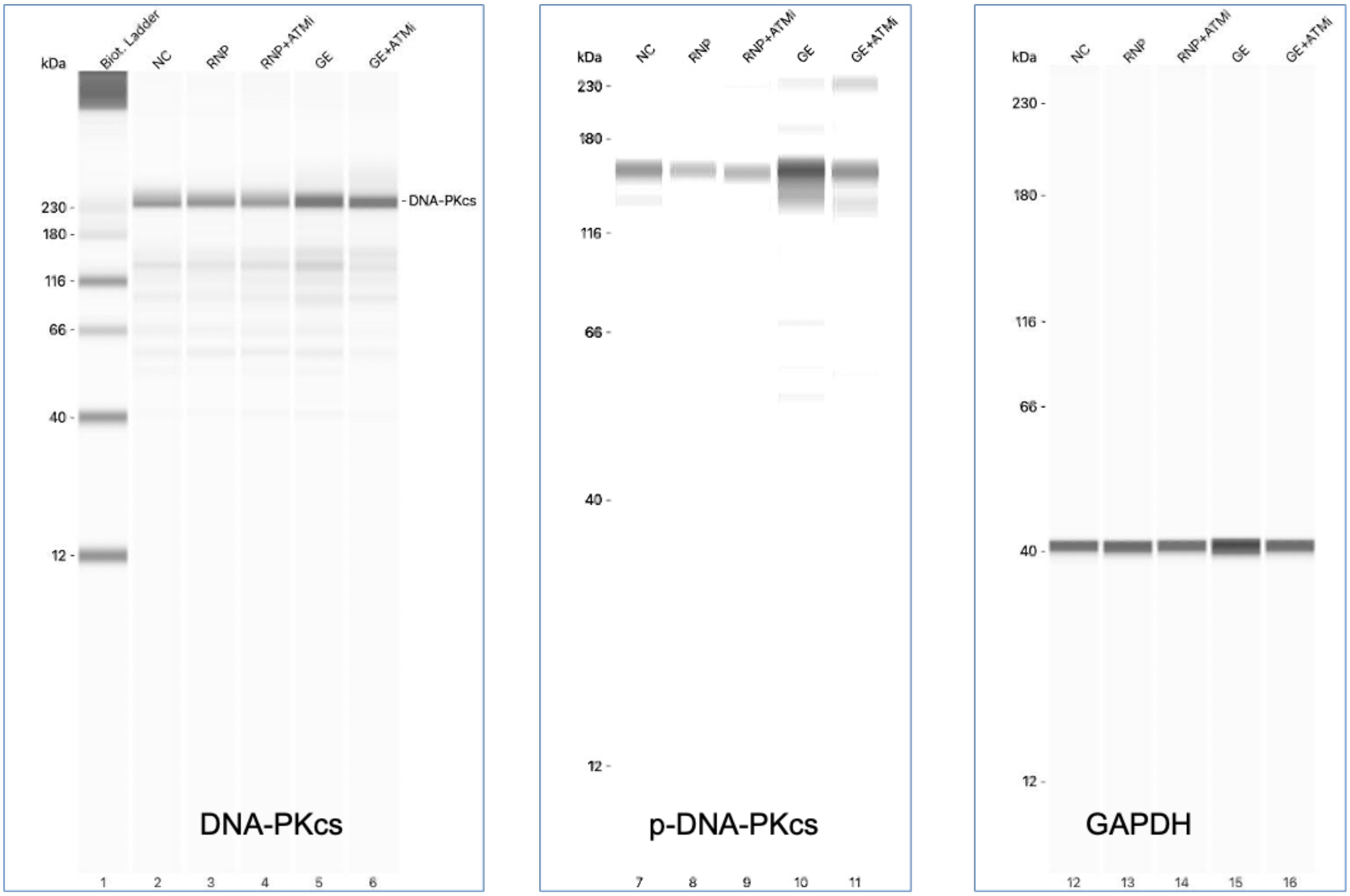

Supplementary Fig. 26| Full, uncropped Simple Western images. The original Simple Western images corresponding to the cropped panel shown in Fig. 7a are presented.

Supplementary Table 1. Intron sequence for pActb-lacZ-mCherry construction

Sequence (5'-3')
TTACTCTTCCTGAGACAGGTTCTGGAAATGAGGGTGAGCTGCAAATAGAAGAGGACTGGTAGAGTTGGGTGTGA TCGCAGAGGGGTCAGGAAGCAGAGGCTGGAGCTGGACTGTGGGGGAGGGCGAGGTGGGTGAAGGAGAGAAG AGAGTGTATCGTCCAGGAGAAGGGTGAGCTGGGGAAGGAGACTCTCCAGGCTGGAGGTAGGTTGGCCGGGAG CCCATGCCTGAAGTTGCTGGAGGAATGTCTGGAGCTCCGTCAGGTCTCGGGTTTGAGGGTGTAACAGGGGGGA GCCCAGTAGGGGGAGTGCTGTGAACATGGGGAGCCGCCGGGGCCCTCGCCCAGGGCGCCGCCCTTGGGGCTG ATCTCGGACACGTGGCACAGCCGCTCGGAGTAGTCTGGCTGCAGGCTCTCAGCCAGGCCCTTGCTCACGCCGG ACCAGGCGCTGAAGTTGCCGTGGTACTCGGTCACCAGGTCCTCCAGGTTCTTCAGGGTTGGGATGCGGGGCATG GTTCTCTCCAGCCAGCAGTACACGCACATCAGGCCCACGATCAGGCCCATGGATCCCAGTGGGATCAGCACGG CCTCCAGAGCGAACAGCAGTGGGTTCTCCTTGCTGGTGTTGCCCCAGTGGATGGGGTGGGACCAGTCGCTCCAC CTCTGGGCGGATCCGCACAGTGGGTTGTACCTGCTCCGCACGCGGAAGGTGTACAGCTTCTGGGCGTCCACGCT TGGCAGGGAGAAGCTCTGCCTGTGGTCCACGGACTGCTCGGTCCAGCTTCTGTCCCTGTCGGAGCGGTACTGCA CCAGGTGCTCCAGGCAGTGGTCCAGGTATCTGTTGGACCAGCTCAGCTCCAGCTGGGACTCGCTCAGGGTTCTC AGGGTCAGGTTAGCTGGAGCCCATGGGATGACCAGGTCCTGCAGCTTCAGGGTCTGTGGGTCCTGCCGGCGTG GCTCCCTTGGGTCCTGCAGCTGCACCACGAAGGTCTGGTACAGCCGGATCTCCTCCTTGCCGAACCAGCAGCCG CTGGTGATGCCCTCGGAGAACAGGTAGTGGCCGCACTCCTGCACCTTGTCGTCGTTGGAGGTCTTGTACCAGTA GTGCAGGGTCAGGTTGGTTGGCTGCAGCTCGGAGCTGGAGTTCCAGGTGCAGTTCATGTACTCCACGTTGAACA CGAAGCACTGCACCTTTGGCAGGGGCAGGGTGGACACGTTCAGGGTGCCTGGTGGGGTGCTCAGCAGCAGGA AGTCAGCGGTGATGTCCTCGTTGCCGCTGTGGGTGAGGACCTTAGGATTGAGGCCGACGCCGAGCAGAGGGAG CTGGAGGAAGAGGAGAGACTTGAC

Supplementary Table 2. The list of the inhibitors that were used in this study except drug screening

No	Name of the reagent	Target	Manufacturer	Cat#	Concentration (μM)	
					For plasmid donor	For AAV do nor
1	NU7441	DNA-PK	Selleck	S2638	10	-
2	RI-1	RAD51	Selleck	S8077	30	-
3	NMS-P118	PARP-1	Selleck	S8363	30	-
4	VE-821	ATR	Selleck	S8007	10	10
5	Ceralasertib	ATR	Selleck	S7693	1	1
6	4-Octyl Itaconate	KEAP1	Selleck	S5929	300	300
7	AZD1390	ATM	Selleck	S8680	0.01	0.01
8	AZ31	ATM	Selleck	S8556	1	1
9	PFTμ	TP53	Selleck	S2930	-	10

Supplementary Table 3. The list of assay modules, reagents and consumables for JESS simple western

No	Name	Manufacturer	Cat#
1	EZ Standard Pack 1 12-230 kDa	Bio-techne	PA-AT01EZ-8
2	EZ Standard Pack 3 66-440 kDa	Bio-techne	PA-AT03EZ-8
3	EZ Standard Pack 5 2-40 kDa	Bio-techne	PA-AT05EZ-8
4	Anti-Rabbit Detection Module	Bio-techne	DM-001
5	Anti-Mouse Detection Module	Bio-techne	DM-002
6	12-230 kDa Fluorescence Separation Module	Bio-techne	SM-FL004
7	66-440 kDa Fluorescence Separation Module	Bio-techne	SM-FL005
8	2-40 kDa Fluorescence Separation Module	Bio-techne	SM-FL003
9	Protein Normalization Module	Bio-techne	DM-PN02
