# Supplementary material for: ATM Inhibition Enhances Knock-in Efficiency by Suppressing AAV-Induced Activation of Apoptotic Pathways
Source: Commun Biol. 2026 Feb 6;9:177. doi: 10.1038/s42003-026-09604-z (PMC12881585; doi:10.1038/s42003-026-09604-z)
Supplement: Supplementary file 3 — Description of Additional Supplementary files [file 42003_2026_9604_MOESM3_ESM.pdf]

Description of Additional Supplementary files

File name: Supplementary Data 1

Description: The list of the reagents in the DNA Damage/ DNA Repair Compound Library

File name: Supplementary Data 2

Description: The list of primers and oligos for plasmid construction

File name: Supplementary Data 3

Description: The list of primers
