# Supplementary material for: ATM Inhibition Enhances Knock-in Efficiency by Suppressing AAV-Induced Activation of Apoptotic Pathways
Source: Commun Biol. 2026 Feb 6;9:177. doi: 10.1038/s42003-026-09604-z (PMC12881585; doi:10.1038/s42003-026-09604-z)
Supplement: Supplementary file 7 — Reporting Summary [file 42003_2026_9604_MOESM7_ESM.pdf]

nature portfolio

Corresponding author(s): Yutaka Hanazono
Hiromasa Hara

Last updated by author(s): Dec 19, 2025

Reporting Summary

Nature Portfolio wishes to improve the reproducibility of the work that we publish. This form provides structure for consistency and transparency in reporting. For further information on Nature Portfolio policies, see our Editorial Policies and the Editorial Policy Checklist.

Statistics

For all statistical analyses, confirm that the following items are present in the figure legend, table legend, main text, or Methods section.

n/a	Confirmed	
☐	☒	The exact sample size (*n*) for each experimental group/condition, given as a discrete number and unit of measurement
☐	☒	A statement on whether measurements were taken from distinct samples or whether the same sample was measured repeatedly
☐	☒	The statistical test(s) used AND whether they are one- or two-sided<br>*Only common tests should be described solely by name; describe more complex techniques in the Methods section.*
☐	☒	A description of all covariates tested
☐	☒	A description of any assumptions or corrections, such as tests of normality and adjustment for multiple comparisons
☐	☒	A full description of the statistical parameters including central tendency (e.g. means) or other basic estimates (e.g. regression coefficient) AND variation (e.g. standard deviation) or associated estimates of uncertainty (e.g. confidence intervals)
☐	☒	For null hypothesis testing, the test statistic (e.g. *F*, *t*, *r*) with confidence intervals, effect sizes, degrees of freedom and *P* value noted<br>*Give P values as exact values whenever suitable.*
☐	☒	For Bayesian analysis, information on the choice of priors and Markov chain Monte Carlo settings
☐	☒	For hierarchical and complex designs, identification of the appropriate level for tests and full reporting of outcomes
☐	☒	Estimates of effect sizes (e.g. Cohen's *d*, Pearson's *r*), indicating how they were calculated

Our web collection on statistics for biologists contains articles on many of the points above.

Software and code

Policy information about availability of computer code

Data collection	FCM: Cell Sorter Software ver2.1.6<br>Simple Western: Compass SW software v7.0.0
Data analysis	Statistical analysis: Prism v10 (GraphPad Software)<br>Image processing: ImageJ v1.53a (NIH; https://imagej.nih.gov/ij/)

For manuscripts utilizing custom algorithms or software that are central to the research but not yet described in published literature, software must be made available to editors and reviewers. We strongly encourage code deposition in a community repository (e.g. GitHub). See the Nature Portfolio guidelines for submitting code & software for further information.

Data

Policy information about availability of data

All manuscripts must include a data availability statement. This statement should provide the following information, where applicable:

- Accession codes, unique identifiers, or web links for publicly available datasets
- A description of any restrictions on data availability
- For clinical datasets or third party data, please ensure that the statement adheres to our policy

The raw deep-sequencing reads generated in this study have been deposited in the NCBI Sequence Read Archive (PRJNA1390995). All other data supporting the findings of this study have been deposited in Zenodo (DOI: 10.5281/zenodo.17982883). Uncropped PCR and blot images are provided as Supplementary

Information. The gating strategies for all flow cytometry plots are also provided in the Supplementary Information. Newly generated plasmids and all other materials are available from the corresponding author upon reasonable request.All data supporting the findings of this study, including uncropped PCR and blot images as well as numerical source data for graphs, are provided in Supplementary Data 1. Additional information is available from the corresponding author upon reasonable request.

Research involving human participants, their data, or biological material

Policy information about studies with human participants or human data. See also policy information about sex, gender (identity/presentation), and sexual orientation and race, ethnicity and racism.

Reporting on sex and gender	This study did not involve human participants, their data, or biological material.
Reporting on race, ethnicity, or other socially relevant groupings	This study did not involve human participants, their data, or biological material.
Population characteristics	This study did not involve human participants, their data, or biological material.
Recruitment	This study did not involve human participants, their data, or biological material.
Ethics oversight	This study did not involve human participants, their data, or biological material.

Note that full information on the approval of the study protocol must also be provided in the manuscript.

Field-specific reporting

Please select the one below that is the best fit for your research. If you are not sure, read the appropriate sections before making your selection.

☒ Life sciences ☐ Behavioural & social sciences ☐ Ecological, evolutionary & environmental sciences

For a reference copy of the document with all sections, see nature.com/documents/nr-reporting-summary-flat.pdf

Life sciences study design

All studies must disclose on these points even when the disclosure is negative.

Sample size	We did not perform a formal sample-size calculation. Sample sizes were chosen based on previous publications demonstrating adequate power for detecting similar effects.
Data exclusions	No data were excluded from the analyses.
Replication	All experiments were performed in three independent replicates, except for the experiment shown in Supplementary Fig. 7b.
Randomization	This was an in vitro study using cultured reporter cells; no random allocation was performed.
Blinding	Blinding was not performed, as all data collection (flow-cytometric quantification of fluorescent reporters, Western blot imaging) and analysis were conducted using automated instruments and predefined, objective thresholds. This approach minimizes subjective bias without the need for manual group-identity masking.

Reporting for specific materials, systems and methods

We require information from authors about some types of materials, experimental systems and methods used in many studies. Here, indicate whether each material, system or method listed is relevant to your study. If you are not sure if a list item applies to your research, read the appropriate section before selecting a response.

Materials & experimental systems

n/a	Involved in the study	
☐	☒	Antibodies
☐	☒	Eukaryotic cell lines
☒	☐	Palaeontology and archaeology
☒	☐	Animals and other organisms
☒	☐	Clinical data
☒	☐	Dual use research of concern
☒	☐	Plants

Methods

n/a	Involved in the study	
☒	☐	ChIP-seq
☐	☒	Flow cytometry
☒	☐	MRI-based neuroimaging

Antibodies

Antibodies used	ATR (E1S3S) Rabbit mAb Cell signaling 1394<br>Phospho-ATR (Ser428) Antibody Cell signaling 2853<br>ATM (D2E2) Rabbit mAb Cell signaling 2873<br>Anti-ATM (phospho S1987) Abcam ab315019<br>Chk1 (2G1D5) Mouse mAb Cell signaling 2360<br>Human/Mouse/Rat Phospho-Chk1 (S317) Antibody RnD systems AF2054<br>NRF2 (D1Z9C) XP® Rabbit mAb Cell signaling 12721<br>p53 (1C12) Mouse mAb Cell signaling 2524<br>Phospho-p53 (Ser15) Antibody Cell signaling 9284<br>Caspase-3 Antibody Cell signaling 9662<br>Cleaved Caspase-3 (Asp175) Antibody Cell signaling 9661<br>DNA-PKcs Polyclonal antibody Proteintech 28534-1-AP<br>Phospho-DNA-PK (Thr2609) Polyclonal Antibody Invitrogen PA5-105749<br>β-Tubulin Antibody Cell signaling 2146<br>GAPDH Antibody (6C5) Santa Cruz sc-32233<br>Peroxidase AffiniPure Donkey Anti-Mouse IgG (H+L) Jackson Immuno Research Laboratories, Inc. 715-035-150<br>Peroxidase AffiniPure Donkey Anti- Rabbit IgG (H+L) Jackson Immuno Research Laboratories, Inc. 715-035-152<br>Alexa Fluor® 647 Annexin V BioLegend 640912
Validation	All primary antibodies were validated by the manufacturer for the indicated applications (Western blot, immunofluorescence).

Eukaryotic cell lines

Policy information about cell lines and Sex and Gender in Research

Cell line source(s)	Mouse embryonic stem cells (male; C57BL/6J background) were kindly provided by Professor Takeshi Yagi at Osaka University.<br>HEK293T cells (female) were obtained from ATCC.
Authentication	None of the cell lines used were formally authenticated.
Mycoplasma contamination	Cell lines were not tested for mycoplasma contamination.
Commonly misidentified lines<br>(See ICLAC register)	No commonly misidentified or cross-contaminated cell lines were used in this study.

Plants

Seed stocks	This study did not involve any plant materials.
Novel plant genotypes	This study did not involve any plant materials.
Authentication	This study did not involve any plant materials.

Flow Cytometry

Plots

Confirm that:

☒ The axis labels state the marker and fluorochrome used (e.g. CD4-FITC).

☒ The axis scales are clearly visible. Include numbers along axes only for bottom left plot of group (a 'group' is an analysis of identical markers).

☒ All plots are contour plots with outliers or pseudocolor plots.

☒ A numerical value for number of cells or percentage (with statistics) is provided.

Methodology

Sample preparation	For reporter ES cells, cells were washed with PBS(–) and detached using 1× TrypLE™ Express solution. After incubation for 5 min at 37 °C, cells were collected in DMEM containing 10% FBS. The collected cells were centrifuged at 300 × g for 3 min. Cell pellets were washed once with PBS(–) and resuspended in PBS(–) containing 7-AAD for viability analysis. Cells were filtered

through a cell strainer and kept on ice prior to flow cytometric analysis.

For HEK293T, cells were washed with PBS(–) and detached using 1× TrypLE™ Express solution. After incubation for 5 min at 37 °C, cells were collected in DMEM containing 10% FBS. The collected cells were centrifuged at 300 × g for 3 min. Cell pellets were washed once with PBS(–) and resuspended in PBS(–) containing DAPI for viability analysis. Cells were filtered through a cell strainer and kept on ice prior to flow cytometric analysis.

For mouse MSCs, cells were washed with PBS(–) and detached using 1× TrypLE™ Express solution. After incubation for 5 min at 37 °C, cells were collected in DMEM containing 10% FBS. The collected cells were centrifuged at 300 × g for 3 min. Cell pellets were washed once with PBS(–) and resuspended in PBS(–) containing DAPI for viability analysis. Cells were filtered through a cell strainer and kept on ice prior to flow cytometric analysis.

For apoptosis assay, cells were washed with PBS(–) and detached using 1× TrypLE™ Express solution. After incubation for 5 min at 37 °C, cells were collected in DMEM containing 10% FBS. The collected cells were centrifuged at 300 × g for 3 min. Cell pellets were washed once with PBS(–) and stained with 200 μL of Zombie NIR for 15 min at room temperature, followed by incubation in 200 μL of Annexin V Binding Buffer containing Alexa Fluor 647–Annexin V 10 μL, for 15 min at room temperature. Cells were filtered through a cell strainer and kept on ice prior to flow cytometric analysis.

Instrument

SH800

Software

Cell Sorter Software

Cell population abundance

For each sample, between 10,000 and 30,000 7-AAD–negative (live) cell events were recorded and analyzed on the SH800 cell sorter.

Gating strategy

For reporter ES cells, cells were first gated on FSC-A and SSC-A to exclude debris and select the main cell population. Single cells were then identified by gating on FSC-W and FSC-H. Live cells were defined as 7-AAD$^-$ events. Within the live singlet population, cells were separated into TagBFP$^-$ and TagBFP+ fractions; the TagBFP-negative fraction was defined as DSB cells. Reporter outcomes were then quantified within the live cell gate based on mEGFP and mCherry fluorescence: mEGFP$^+$/mCherry$^+$ cells were classified as knock-in, whereas mEGFP$^+$/mCherry$^-$ cells were classified as EJ-TI.

For HEK293T, cells were first gated on FSC-A and SSC-A to exclude debris and select the main cell population. Single cells were then identified using FSC-W and FSC-H. Live cells were defined as DAPI$^-$ events. Within the live singlet population, mEGFP+ cells were quantified and classified as knock-in cells.

For mouse MSCs, cells were first gated on FSC-A and SSC-A to exclude debris and select the main cell population. Single cells were then identified using FSC-W and FSC-H to remove doublets. Live cells were defined as DAPI$^-$ events. Within the live singlet population, mEGFP+ cells were quantified and classified as knock-in cells.

For apoptosis assay, cells were first gated on FSC-A and SSC-A to exclude debris and select the main cell population. Single cells were then identified using FSC-W and FSC-H. Live cells were defined as Zombie NIR$^-$ events. Apoptotic cells were then quantified within the live cell gate based on Annexin V-AF647 and mCherry fluorescence: Annexin V-AF647$^+$/mCherry$^+$ cells were classified and quantified as apoptotic cells.

☒ Tick this box to confirm that a figure exemplifying the gating strategy is provided in the Supplementary Information.
